# The Proper Light and Arrangement of the Dental Operating Rooms

**Published:** 1906-12-15

**Authors:** George B. Sanford


### THE PROPER LIGHT AND ARRANGEMENT OF THE DENTAL OPERATING ROOMS.

BY GEORGE B. SANFORD, D.D.S.

Beginners, and others in the dental profession, seem to have very erroneous ideas as to the arrangement of their operating rooms, and they seem to remain content with such facilities as may be obtained in such rooms as may be in the location of the place they have selected for operating, and are often discouraged with the apparent hopelessness of turning such natural surroundings into anything of modern convenience for this purpose.

The facts are, that buildings, modern and otherwise, seem to be constructed for any or all other purposes than dental operating rooms. In addition to plenty of water near at hand, what is desirable as a standard to work from in operating rooms is good sunlight; north if possible, but plenty of it, which can be regulated by methods mentioned later on.

This light is often rendeerd destructive to the best eyesight by reflection from glass windows on the opposite side of the street, and it is impossible to avoid this condition with any operating chair that remains immovable laterally in the window space in which it is set.

Sun and other light is variable and will be from the most intense glare to semi-darkness and will require frequent shifting of the chair laterally in the window space to obtain the most favorable condition.

Now, how can these conditions be the most readily brought about? The floor in the operating room, and especially under the operating chairs should always be free from carpeting. If it is soft pine, stop up the cracks with putty and go over it with one or two coats of thin shellac, in alcohol, then dissolve bee's-wax in turpentine; go over it with this, wiping off the excess and you will have an antiseptic floor. If hard wood, use linseed oil, with a small amount of burnt umber, or other coloring matter for the first coat,

Now, having the floor in this clean or antiseptic condition (and this treatment applies equally well to the laboratory), you can easily keep it clean by shifting the chair, cabinet or work bench in any relative position you desire. Shifting the chair one for the most favorable light will be only one of the purposes in which these conditions will be useful. Others not so apparent will develop when you have adopted method.

I have thus far neglected to say that all your furniture should be placed on casters—casters that are not constantly out of order, and will move with the furniture freely, in any direction on the floor.

Cabinet furniture manufacturers are in the habit of supplying the cheapest caster they can obtain, with the result of selling a worthless article at an exorbitant price—*Dental Times*.

